# A High-Throughput Automated Microfluidic Platform for Calcium Imaging of Taste Sensing

**DOI:** 10.3390/molecules21070896

**Published:** 2016-07-08

**Authors:** Yi-Hsing Hsiao, Chia-Hsien Hsu, Chihchen Chen

**Affiliations:** 1 Institute of Nanoengineering and Microsystems, National Tsing Hua University, Hsinchu 30013, Taiwan; s102035802@gmail.com (Y.-H.H.); chsu@nhri.org.tw (C.-H.H.); 2 Institute of Biomedical Engineering and Nanomedicine, National Health Research Institutes, Miaoli 35053, Taiwan; 3 Department of Power Mechanical Engineering, National Tsing Hua University, Hsinchu 30013, Taiwan

**Keywords:** automated system, microfluidic, high throughput, calcium imaging, single cell, glucose, sucralose, denatonium benzoate, enteroendocrine cell, taste cell

## Abstract

The human enteroendocrine L cell line NCI-H716, expressing taste receptors and taste signaling elements, constitutes a unique model for the studies of cellular responses to glucose, appetite regulation, gastrointestinal motility, and insulin secretion. Targeting these gut taste receptors may provide novel treatments for diabetes and obesity. However, NCI-H716 cells are cultured in suspension and tend to form multicellular aggregates, preventing high-throughput calcium imaging due to interferences caused by laborious immobilization and stimulus delivery procedures. Here, we have developed an automated microfluidic platform that is capable of trapping more than 500 single cells into microwells with a loading efficiency of 77% within two minutes, delivering multiple chemical stimuli and performing calcium imaging with enhanced spatial and temporal resolutions when compared to bath perfusion systems. Results revealed the presence of heterogeneity in cellular responses to the type, concentration, and order of applied sweet and bitter stimuli. Sucralose and denatonium benzoate elicited robust increases in the intracellular Ca^2+^ concentration. However, glucose evoked a rapid elevation of intracellular Ca^2+^ followed by reduced responses to subsequent glucose stimulation. Using *Gymnema sylvestre* as a blocking agent for the sweet taste receptor confirmed that different taste receptors were utilized for sweet and bitter tastes. This automated microfluidic platform is cost-effective, easy to fabricate and operate, and may be generally applicable for high-throughput and high-content single-cell analysis and drug screening.

## 1. Introduction

The gustatory system of mammals can distinguish thousands of substrates when a substrate in the oral cavity biochemically binds to taste receptors in taste bud cells [1,2]. Interestingly, taste receptors exist not only on the tongue but also on other organs and tissues of the human body [3,4,5]. For example, sweet taste receptors are expressed in human gut cells and they can sense tastes using the same mechanism as the human tongue [5,6,7]. Evidence has suggested that sweet taste receptors expressed in L cells, a type of enteroendocrine cell, play important roles in diabetes and obesity [7,8]. The activation of sweet taste receptors by glucose in gut cells is known to elevate the concentration of intracellular Ca^2+^ ions, which has been hypothesized to regulate the secretion of glucagon-like peptide-1 (GLP-1) [9,10,11,12,13]. GLP-1 may, in turn, cause the subsequent release of insulin [8,14,15,16]. Modulating the secretion of GLP-1 molecules from “taste cells” in the gut may provide an important model for sweet taste stimulation, especially on meal-induced insulin secretion [14,17,18,19]. Rational drug design has been targeting sweet taste receptors to control natural signal transduction and reduce glucose concentration in the blood, since the current widely used medicine for the treatment of type II diabetes, Metformin, may cause side effects, such as lactic acidosis, nausea, and abdominal pain in patients [20]. However, mechanisms by which glucose uptake and insulin secretion are regulated are not yet fully understood.

The human enteroendocrine L cell line NCI-H716, containing both sweet and bitter taste receptors, as well as G-protein subunit α-gustducin and several other testate transduction elements, constitutes a unique model to study the secretion of GLP-1 molecules [5,18,19,21,22,23,24,25]. Its release of GLP-1 is stimulated by sugars and artificial sweeteners, such as sucralose. Interestingly, binding of bitter tastants to bitter taste receptors also induces rises in intracellular Ca^2+^ and the release of GLP-1 [26,27]. In contrast, it is found that sweet and bitter taste receptors are typically expressed in segregated cells in the oral cavity [28], while sweet tastes predict food and bitter tastes signal for toxic compounds. It has been suggested bitter compounds–induced secretion of GLP-1 may function as a means of host defense to delay gastric emptying and lessen substance ingestion [23]. It remains to be comprehensively deciphered how the information is processed upon sweet and bitter taste activation in enteroendocrine L cells [14,29,30].

In this study, we focused on studying Ca^2+^ responses and the potential roles of NCI-H716 cells in diabetes. Calcium imaging, offering real-time and high-content images, is widely utilized for studying cellular signal transduction upon stimulation. However, NCI-H716 cells are maintained in suspension culture and are prone to aggregation, which precludes high-throughput calcium imaging on single cells in conventional bath and perfusion systems. Profiling physiological responses of single cells to extracellular stimuli is critical. Traditional approaches are limited by measuring responses of an ensemble of cells, which overlooks heterogenetic behaviors within single cells. Microfluidic tools have been developed to trap single cells [31,32,33] and to perform calcium imaging on cells, tissues, or nematodes [34,35,36,37,38]. Here, we integrated an automated fluidic control unit combined with a microfluidic chip that is capable of loading, trapping, staining, and chemically stimulating and recording from an array of single cells. In contrast to conventional systems, which typically consume more than 500 μL of a solution for detecting Ca^2+^ signals for each treatment condition, our platform requires only 2 μL. This automation platform provides a simple, rapid, and reliable means to study Ca^2+^ signal transduction upon chemical stimuli of various concentrations.

## 2. Results

### 2.1. Trapping and Perfusion of NCI-H716 Cells

**Figure 1:**
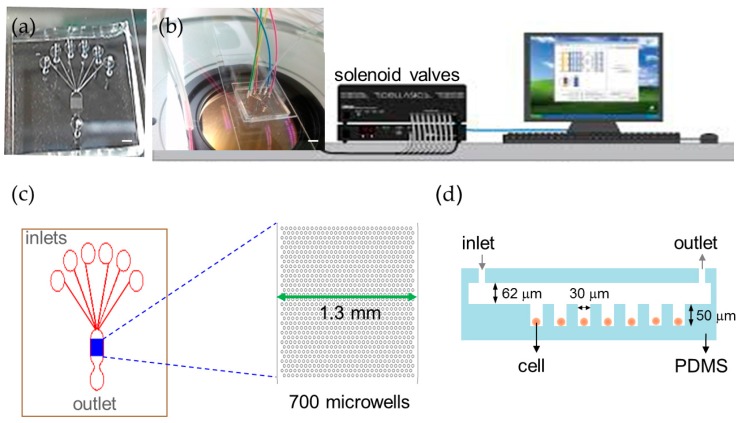
Automatic microfluidic platform for calcium imaging. (**a**) Photograph of the microfluidic chip (scale bar = 1 mm); (**b**) Experimental setup of the automated platform with the microfluidic chip and solenoid valves (scale bar = 2 mm); (**c**) The microfluidic chip contains 700 microwells that are 30 μm in diameter to array cells and six inlets to perfuse different solutions in a single experiment; (**d**) A cross-section schematic of the microfluidic device.

**Figure 2:**
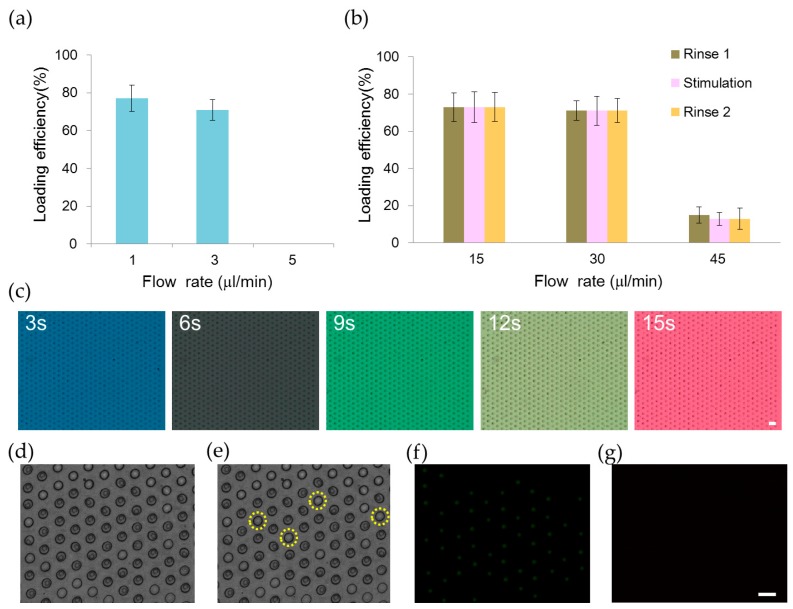
Loading and perfusion of human L cell line NCI-H716 cells in the microfluidic chip. (**a**) Loading efficiency of NCI-H716 cells into microwells at different flow rates controlled by using a syringe pump; (**b**) Relationship between the loading efficiency and the flow rate of the perfusion solution. Three solutions (rinse 1, stimulation, rinse 2) were introduced into the device sequentially. The flow rate of 15, 30, and 45 μL/min was generated by applying compressed air of pressure 5, 10, and 15 psi to the inlet, respectively; (**c**) Five different dye solutions are introduced sequentially into the microfluidic chip by applying a pressure of 10 psi to the inlet. Micrographs are taken at every 3 s; A sequence of micrographs showing (**d**) NCI-H716 cells that are loaded into microwells; (**e**) Four cells that are dislodged after rinsing with a buffer solution; (**f**) Live and (**g**) dead cell staining of NCI-H716 cells after one day of incubation. Live cells appear green while dead cells appear red under the fluorescence microscope. Scale bar = 50 μm.

We developed an automation platform integrating a microfluidic chip and solenoid valves. The microfluidic chip comprised a main channel incorporating an array of 700 microwells on its floor, one outlet, and six inlets for introducing cells, reagents, and different chemical stimuli (Figure 1). The main channel was 1.3 mm in width, 62 μm in depth and 2.6 mm in length. The microwell was 30 μm in diameter, 50 μm in depth and 30 μm in spacing. We characterized the loading efficiency by introducing NCI-H716 cells at 1 × 10^6^ cells/mL concentration into the microfluidic chip. The cell-loading efficiency was improved by using the automated fluid control and by tailoring dimensions of the microwell for NCI-H716 cells. More than 500 single cells were entrapped into microwells in 2 min at a flow rate of 1 μL/min, corresponding to a loading efficiency of 77% (Figure 2a). The loading efficiency decreased with the increased flow rate and decreased to zero when a flow rate of 5 μL/min was applied. Solutions were exchanged by applying a pressure to the inlet. Cells remained trapped when the applied pressure was smaller than 10 psi, and were largely dislodged when the pressure was 15 psi (Figure 2b). Solutions inside the microchip were successfully exchanged within every 3 s as shown in Figure 2c. Figure 2d–g show a sequence of micrographs taken after loading, perfusion, and live and dead staining of NCI-H716 cells, respectively. The viability of NCI-H716 cells after one day of incubation was approaching 100%, as indicated by the live/dead staining results.

### 2.2 Stimulation of NCI-H716 Cells with Sweet Tastants

**Figure 3:**
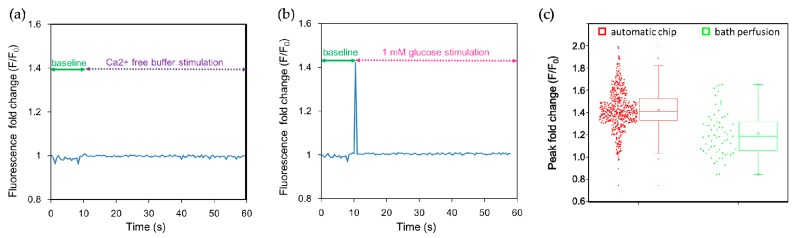
Human L cell line NCI-H716 cells were stimulated in the automated microfluidic chip with the sweet tastant. (**a**) Ca^2+^-free buffer was introduced as a control; no peak in the fluorescence fold change was observed after stimulation; (**b**) Then 1 mM glucose solution was introduced at 10 s, and a peak in the fluorescence intensity associated with an increase of intracellular calcium concentration rapidly appeared; (**c**) Peak fold changes in the fluorescence intensity indicate heterogeneity in calcium responses of single cells. The average peak fold change was 1.39 obtained using the automated platform (*n* = 1382) and 1.21 obtained using the bath perfusion system (*n* = 64), respectively. The difference in peak fold changes was significant (*p* < 0.001) as determined by the two-tailed Student’s *t*-test. Cohen’s effect size value (*d* = 0.65) suggested a moderate to high practical significance.

Many tastes, including sweet and bitter stimuli, elicit Ca^2+^ release from internal stores and/or promote Ca^2+^ entry [26,27]. We therefore used calcium imaging to monitor cellular responses. Intracellular Ca^2+^ concentrations were quantified using the calcium indicator dye Fluo-4. Fluo-4 dyes are single-wave calcium probes (excitation around 490 nm, emission around 520 nm; non-ratiometric measurement) whose emission intensity depends on the amount of calcium bound, i.e., an increase in the amount of calcium results in an increase in fluorescence signal brightness. When NCI-H716 cells were stimulated with Ca^2+^-free buffer solution, no obvious fluorescence response was observed (Figure 3a). On the contrary, a 1 mM glucose solution elicited an abrupt increase in the fluorescence intensity observed at 0.5 s after stimulation, indicating a rapid increase in their intracellular calcium concentration (Figure 3b). The narrow width of the peak may be due to a recording from a single cell, and the rapid re-establishment of the low intracellular calcium level by the sequestration of Ca^2+^ to the endoplasmic reticulum and the activation of the transient receptor potential cation channel subfamily M member 5 (TRPM5) [39,40]. To quantify the augmentation in the fluorescence intensity, we normalized the fluorescence intensity (*F*) with respect to the baseline fluorescence signal (*F*_0_) measured for 10 s before the stimulation; this signal can be expressed as *F*/*F*_0_, which represents a fold change of the fluorescence intensity after stimulation. We observed that the calcium responses of individual cells exhibit distinct cell heterogeneity, as shown in Figure 3c and summarized in Table 1. The peak fold change of 1382 cells measured using the automated chip and 64 cells using bath perfusion was 1.39 ± 0.27 folds and 1.21 ± 0.20 folds, respectively. The difference in the peak fold change was significant as suggested by the small *p*-value (*p* < 0.001) and moderate Cohen’s effective size value (*d* = 0.65). The range and the quartile coefficient of dispersion in the peak fold change were further compared (Table 1), indicating that the calcium imaging responses observed using the automated platform spanned a wider range, but were also more congregated around the median. In contrast, bath perfusion approaches are restricted by the determination of responses from a small number of cells. In summary, we have developed a simple microfluidic platform to investigate intracellular calcium responses to dynamic conditions after stimulation in real time. This apparatus is simple to operate and eliminates the need for a complex process to achieve high-throughput calcium imaging.

### 2.3 Order of Application of Tastant Stimuli

**Figure 4:**
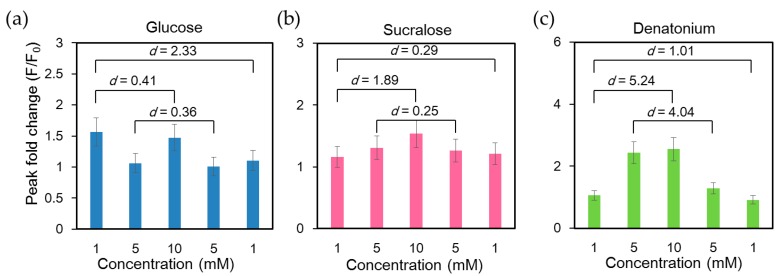
Analysis of intracellular Ca^2+^ response of single human L cell line NCI-H716 cells loaded with Fluo-4 calcium indicator and stimulated with glucose, sucralose, and denatonium benzoate solutions of different concentrations consecutively. Cells were rinsed with a calcium-free basal salt solution between different stimuli. The difference between any two data sets was significant (*p* < 0.001), since a sufficiently large number of cells were assessed (*n* = 1127). Cohen’s *d* values between peak fold changes under stimuli of the same concentrations as well as the lowest and highest concentrations were labeled; (**a**) A large Cohen’s effect size value (*d* = 2.33) suggested a high practical significance between the two 1 mM glucose stimuli; (**b**) Cohen’s effect size values of 0.25 and 0.29 suggested small differences between the two 1 mM and two 5 mM sucralose stimuli. A large Cohen’s effect size value (*d* = 1.89) suggested a high practical significance between 1 mM and 10 mM sucralose stimuli; (**c**) Large Cohen’s *d* values implied that NCI-H716 cells responded to both the order and concentration of denatonium stimuli.

Functions of taste receptors in the gastrointestinal tract involve signal transmission, releasing gut hormones to regulate the homeostasis of energy and glucose via nutrient uptake and neurotransmitter release. Therefore, they play an important role in the communication between the gastrointestinal tract, epithelial and muscle cells and the brain to regulate gastrointestinal function, food intake and glucose metabolism. The taste receptor TAS1Rs are activated by sugars such as glucose and structurally diverse artificial sweeteners such as sucralose [41]. Bitter taste is sensed by the activation of a different taste receptor, TAS2Rs. We first examined the calcium response of NCI-H716 cells under repetitive cycles of glucose stimulation and buffer rinsing. As shown in Supplementary Materials Figure S1, the intracellular Ca^2+^ response remained stable for at least 10 repeated stimulations with 10 mM glucose (Figure S1). To characterize the calcium response upon the activation of different taste receptors expressed in NCI-H716 cells, we stimulated cells with bitter tastant (denatonium benzoate) and sweet tastants (glucose and sucralose). Glucose, sucralose, and denatonium benzoate solutions with concentrations of 1 mM, 5 mM, 10 mM, 5 mM and 1 mM were introduced into the microchip consecutively. Intracellular calcium exhibited the strongest response at the first glucose stimulation and exhibited reduced responses to subsequent stimulations (Figure 4a). Similar findings have been reported in pancreatic β cells, in which the initial fluorescence peak was more pronounced than the subsequent ones upon glucose stimulation [40,42]. In contrast, the effects of sucralose and denatonium benzoate were concentration-dependent (Figure 4b,c).

### 2.4 Calcium Responses to Glucose and Denatonium Benzoate after Gymnema Sylvestre (GS) Treatment

**Figure 5:**
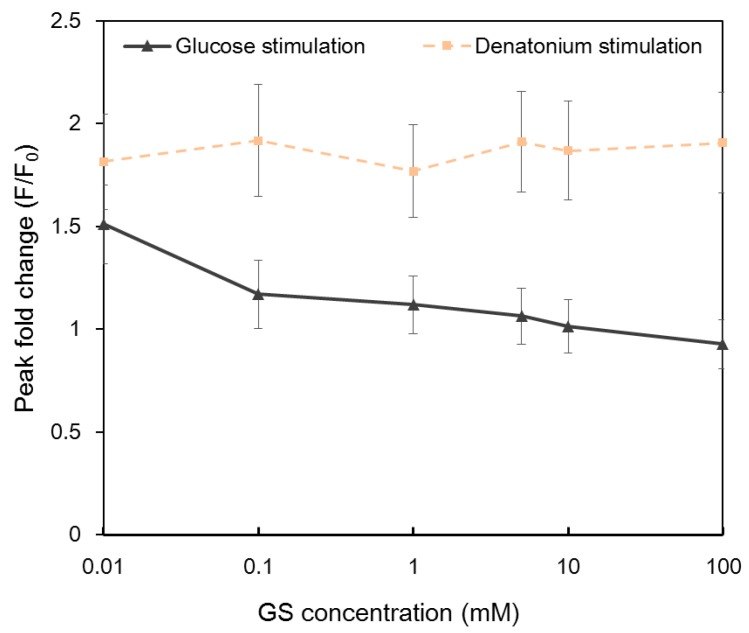
Intracellular Ca^2+^ response of single NCI-H716 cells stimulated with 10 mM glucose and denatonium benzoate after being treated with different concentrations of *Gymnema sylvestre* (GS) solutions. Cells were rinsed with calcium-free basal salt solution between different stimuli. Images were taken from the entire array of cells. A total of 955 cells in two automatic microfluidic chips were imaged.

*Gymnema sylvestre* (GS) is a plant regarded as a potent anti-diabetic agent. It has been reported that gymnemic acids are the active compound of the plant [9,43]. GS competes with sugars for sweet taste receptors and functions as a sweet taste inhibitior. The inhibition is reliable and reversible. Calcium imaging results indicated that the application of GS reduced the intracellular calcium response to glucose in a dose-dependent manner (Figure 5). Intracellular Ca^2+^ responses decreased with the increasing concentration of GS and completely disappeared when the concentration of GS was 100 mM. In contrast, no obvious change was observed in GS-treated NCI-H716 cells to denatonium benzoate, confirming that different taste receptors were utilized for sweet and bitter tastes.

## 3. Discussion

Techniques evaluating the function of endocrine and pancreatic cells are required for developing novel therapies for diseases such as diabetes. One of the major limitations in current techniques is the absence of temporal information, especially related to single cell activation and downstream signal transduction. Recently, single cell analysis with an enhanced temporal resolution has been reported in studies of neural and endocrine physiology [44,45,46,47,48]. Microfluidic tools have been developed for trapping single cells to support high-throughput data acquisition and analysis [49]. Here we extend these approaches and demonstrated an approach to analyze the concentration of Ca^2+^ ions inside single NCI-H716 cells in response to a series of sweet and bitter tastant stimulations. This approach focused on the robustness of the quantification and temporal resolution of calcium imaging. We have developed an automated platform combining a microfluidic chip and solenoid valves for high-throughput monitoring of single cells’ responses to multiple stimuli.

In gustatory taste receptors, TAS1Rs and TAS2Rs are expressed to detect sweet and bitter tastants, respectively. It has been reported that sucralose induces GLP-1 secretion from human L cell line NCI-H716 cells in a concentration-dependent manner, and this secretion of GLP-1 is inhibited by the sweet receptor inhibitor lactisole [50,51]. Similarly, we found the calcium responses upon sucralose and denatonium benzoate activation are concentration-dependent, and blocking the sweet taste receptors using *Gymnema sylvestre* reduces NCI-H716 cells’ cellular response to glucose but not to denatonium benzoate, confirming different taste receptors were utilized for sweet and bitter tastes. Both sweet and bitter tastants stimulated taste receptors on the surface of human L cell line NCI-H716 cells induce an increase in intracellular Ca^2+^ concentration, resulting in the secretion of GLP-1, an important regulator for insulin secretion. Agonists of sweet receptors have been actively investigated as treatments for type 2 diabetes and obesity [52,53,54]. Our results suggest agonists of bitter receptors may play similar roles as agonists of sweet receptors.

## 4. Materials and Methods

### 4.1 Device Fabrication and Operation

The microfluidic chip was made in polydimethylsiloxane (PDMS) and bonded onto a glass slide using a modified soft lithography technique [55]. Briefly, a master for replica molding was created using a photolithographically patterned negative photoresist (SU-8 100, MicroChem, Newton, MA, USA) on a silicon wafer. PDMS pre-polymer (Sylgard 184, Dow Corning, Midland, MI, USA) was poured onto the mold and cured in a conventional oven at 65 °C for 24 h. The cured PDMS replica was removed from the mold and bonded to a glass slide after a brief oxygen plasma treatment (18 W, 1% oxygen, 30 s). A puncher with an inner diameter of 0.75 mm (Harris Uni-Core™, Ted Pella, Redding, CA, USA) was used to punch inlet and outlet holes in the PDMS device. Each inlet was connected via a 35 cm length of Tygon tubing (0.01 inch inner diameter, Small Parts Inc., Longanport, IN, USA) to either a syringe pump (KDS230, KD Scientific, Holliston, MA, USA) or a common regulated compressed air source via a normally closed solenoid valve (S070M-5BG-32, SMC Inc., Tokyo, Japan). The valve was turned on for 3 s for each exchange of solutions. A syringe pump was used to achieve a desired slow flow (<5 μL/min) for loading of cells, while the regulated air was used for the rapid exchange of solutions.

### 4.2 Culture of NCI-H716 Cells

Human enteroendocrine NCI-H716 cells (Bioresource Collection and Research Center, Hsinchu, Taiwan) were routinely maintained in suspension in RPMI 1640 medium supplemented with 10% fetal bovine serum, 2 mM l-glutamine, and 1% penicillin and streptomycin. One day before experiments, cells were loaded into a microfluidic chip and cultured overnight in a humidified incubator at 37 °C with 5% CO_2_.

### 4.3 Stimulation of Tastants and Calcium Imaging on NCI-H716 Cells

Intracellular Ca^2+^ concentrations were quantified using calcium indicator dye Fluo-4. Human L cell line NCI-H716 cells were rinsed twice with a basal salt solution (Wako Pure Chemical Industries Ltd., Osaka, Japan). Cells were subsequently incubated in a calcium-free basal salt solution with 1 mM Fluo-4 (Thermo Fisher Scientific Inc., Waltham, MA, USA) and 10% Pluronic F-127 for 40 min at room temperature in the dark [56]. Thereafter, cells were rinsed with a calcium-free basal salt solution before exposure to each stimulation solution. Tastants, including glucose, sucralose, denatonium benzoate, and *Gymnema sylvestre* extract (GS, Sigma-Aldrich, St. Louis, MO, USA), of various concentrations were prepared in a calcium-free basal salt solution. Cells were imaged on an inverted microscope with an HCX PL APO 10×/0.4 PH1 objective. Fluorescent images were recorded every 0.5 s for 10 s as a baseline and 50 s for each stimulation. Cellular responses from large numbers of cells were analyzed using an imaging software (MetaMorph, Molecular Devices, Sunnyvale, CA, USA). Each cell was selected as a region of interest, and its fluorescence intensity was quantified and subjected to the two-tailed Student’s *t*-test to determine the *p*-values. Effect size was reported using Cohen’s term *d*, determined by the following equation: [mean (group 1) − mean (group 2)]/pooled standard deviation [57]. The deviation may be further quantified using indices, such as the range and the quartile coefficient of dispersion. The quartile coefficient of dispersion, which is the ratio of the interquartile range to the median, was used to make comparisons between data sets.

## 5. Conclusions

We have developed an automated platform that is capable of trapping single cells in microwells within two minutes, delivering multiple chemical stimuli, and recording Ca^2+^ images from single cells. All the experimental processes can be completed with little manual intervention. In contrast to conventional systems, which typically use more than 500 µL of a solution for detecting Ca^2+^ signals in 24-well plates, our platform requires only 2 µL of a stimulation solution for each investigated experimental condition. In addition, this automated microfluidic platform allows precise fluidic control and high-throughput single cell analysis, and enables high-resolution calcium imaging. Calcium imaging results reveal the heterogeneity in cellular responses to glucose, sucralose, and denatonium benzoate stimulation. Sucralose and denatonium benzoate elicit a concentration-dependent response, whereas glucose induces an order-dependent response. Calcium responses to glucose can be blocked by the treatment of the sweet taste inhibitior GS, suggesting that GS might be utilized as a supportive therapy for weight management and diabetes. This automated platform may aid in the single cell analysis for research in fields such as gustatory, diabetes, and obesity.

## Figures and Tables

**Table 1:** Comparison of calcium responses of NCI-H716 cells to 1 mM glucose stimulation in the automatic chip and bath perfusion experiments.

Calcium Imaging Responses	Peak Fold Change	*p*-Value ^1^	Cohen’s Effect Size (*d*)	Range	Quartile Coefficient of Dispersion
Automatic chip (*n* = 1382)	1.39 ± 0.27	<0.001	0.65	4.32	7.7%
Bath perfusion (*n* = 64)	1.21 ± 0.20			0.81	10.7%

^1^ *p*-value was determined by the two-tailed Student’s *t*-test.

## Supplementary Materials

Supplementary materials can be accessed at: http://www.mdpi.com/1420-3049/21/7/896/s1.
