# Supplementary material for: A High-Throughput Automated Microfluidic Platform for Calcium Imaging of Taste Sensing
Source: Molecules. 2016 Jul 8;21(7):896. doi: 10.3390/molecules21070896 (PMC6273845; doi:10.3390/molecules21070896)
Supplement: Supplementary file 1 [file molecules-21-00896-s001.pdf]

Supplementary Materials: A High-Throughput Automated Microfluidic Platform for Calcium Imaging of Taste Sensing

Yi-Hsing Hsiao, Chia-Hsien Hsu and Chihchen Chen

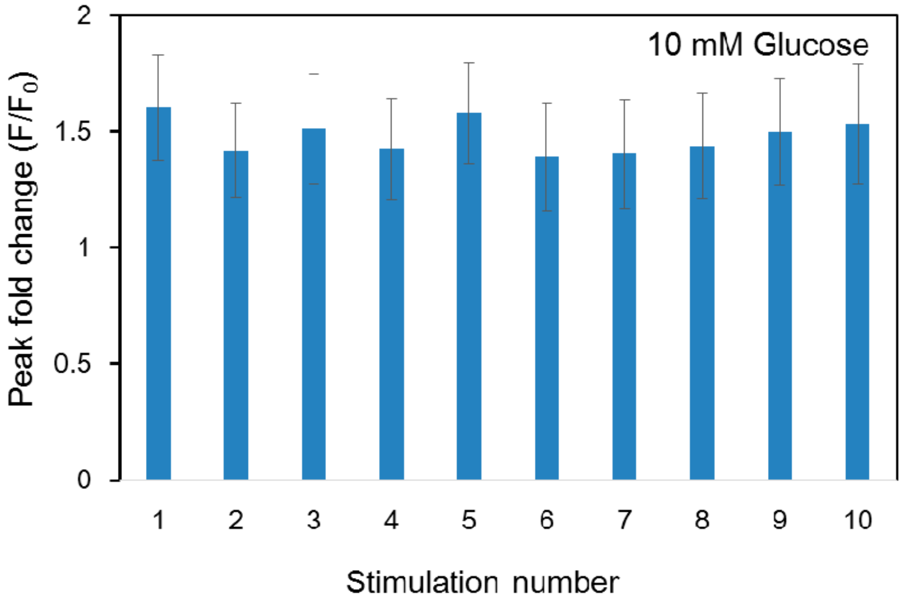

Figure S1. Analysis of intracellular Ca^{2+} response of single human L cell line NCI-H716 cells loaded with Fluo-4 calcium indicator stimulated with 10 repetitive treatments of 10 mM glucose solutions. Cells were rinsed with calcium-free basal salt solution between stimuli (n = 393).
